# Alcohol Abstinence Rescues Hepatic Steatosis and Liver Injury *via* Improving Metabolic Reprogramming in Chronic Alcohol-Fed Mice

**DOI:** 10.3389/fphar.2021.752148

**Published:** 2021-09-16

**Authors:** Aiwen Pi, Kai Jiang, Qinchao Ding, Shanglei Lai, Wenwen Yang, Jinyan Zhu, Rui Guo, Yibin Fan, Linfeng Chi, Songtao Li

**Affiliations:** ^1^ School of Public Health, Zhejiang Chinese Medical University, Hangzhou, China; ^2^ School of Life Science, Zhejiang Chinese Medical University, Hangzhou, China; ^3^ Academy of Chinese Medical Science, Zhejiang Chinese Medical University, Hangzhou, China; ^4^ Molecular Medicine Institute, Zhejiang Chinese Medical University, Hangzhou, China; ^5^ Department of Dermatology, People’s Hospital of Hangzhou Medical College, Zhejiang Provincial People’s Hospital, Hangzhou, China; ^6^ School of Basic Medicine, Zhejiang Chinese Medical University, Hangzhou, China

**Keywords:** alcoholic liver disease, alcohol abstinence, hepatic steatosis, liver injury, hepatic inflammation

## Abstract

**Background:** Alcoholic liver disease (ALD) caused by chronic ethanol overconsumption is a common type of liver disease with a severe mortality burden throughout the world. The pathogenesis of ALD is complex, and no effective clinical treatment for the disease has advanced so far. Prolonged alcohol abstinence is the most effective therapy to attenuate the clinical course of ALD and even reverse liver damage. However, the molecular mechanisms involved in alcohol abstinence-improved recovery from alcoholic fatty liver remain unclear. This study aims to systematically evaluate the beneficial effect of alcohol abstinence on pathological changes in ALD.

**Methods:** Using the Lieber-DeCarli mouse model of ALD, we analysed whether 1-week alcohol withdrawal reversed alcohol-induced detrimental alterations, including oxidative stress, liver injury, lipids metabolism, and hepatic inflammation, by detecting biomarkers and potential targets.

**Results:** Alcohol withdrawal ameliorated alcohol-induced hepatic steatosis by improving liver lipid metabolism reprogramming *via* upregulating phosphorylated 5′-AMP -activated protein kinase (p-AMPK), peroxisome proliferator-activated receptor-α (PPAR-α), and carnitine palmitoyltransferase-1 (CPT-1), and downregulating fatty acid synthase (FAS) and diacylglycerol acyltransferase-2 (DGAT-2). The activities of antioxidant enzymes, including superoxide dismutase (SOD) and glutathione peroxidase (GSH-px), were significantly enhanced by alcohol withdrawal. Importantly, the abstinence recovered alcohol-fed induced liver injury, as evidenced by the improvements in haematoxylin and eosin (H&E) staining, plasma alanine aminotransferase (ALT) levels, and liver weight/body weight ratio. Alcohol-stimulated toll-like receptor 4/mitogen-activated protein kinases (TLR4/MAPKs) were significantly reversed by alcohol withdrawal, which might mechanistically contribute to the amelioration of liver injury. Accordingly, the hepatic inflammatory factor represented by tumour necrosis factor-alpha (TNF-α) was improved by alcohol abstinence.

**Conclusion:** In summary, we reported that alcohol withdrawal effectively restored hepatic lipid metabolism and reversed liver injury and inflammation by improving metabolism reprogramming. These findings enhanced our understanding of the biological mechanisms involved in the beneficial role of alcohol abstinence as an effective treatment for ALD.

## Introduction

Alcoholic liver disease (ALD), one of the main causes of chronic liver disease, ranges from alcoholic fatty liver (AFL) to alcohol hepatitis, alcoholic hepatic fibrosis, alcoholic cirrhosis, and even hepatocellular carcinoma (Seitz et al., 2018). ALD is becoming the prevalent liver disease in the modern world (Friedman et al., 2018; Younossi et al., 2016; O'Shea et al., 2010).

Multiple factors are involved in the progression of ALD, including sex, obesity, and genetics, but how these aspects influence the clinical outcome remains unclear (Becker et al., 1996). Long-term excess alcohol consumption is a predominant etiological factor of ALD progression (Zakhari and Li, 2007). Alcohol abuse causes a disorder in liver lipid metabolism by inducing metabolic reprogramming. The inhibition of β-oxidation and the increase in the *de-novo* biosynthesis of free fatty acids (FFAs) caused by alcohol consumption play an important role in the development of AFL (Ceni et al., 2014). In addition, excessive oxidative stress, hepatocyte apoptosis, and innate immune response are also typical pathological features of ALD (Osna et al., 2017). During the past several decades, much progress has been made, but our understanding of the pathogenesis of ALD remains incomplete.

Prolonged alcohol abstinence is the most effective therapy to attenuate the clinical course of ALD and even reverses liver damage (Seitz et al., 2018; Singal et al., 2018; Forrest et al., 2019). Although from a clinical standpoint, abstinence from alcohol reverses ALD, the molecular mechanisms through which alcohol withdrawal protecting against hepatic steatosis and liver injury are largely unclear. The evidence that can be retrieved showing that alcohol withdrawal restored receptor-mediated endocytosis in rats (Casey et al., 1989), and normalised aberrant fatty acid transport, FFAs oxidation, and restored lysosomal function to attenuate the liver injury (Thomes et al., 2019). In the present study, we systematically examined whether alcohol withdrawal reverses alcohol-induced alterations in lipid metabolism, liver injury, oxidative stress, and inflammation. We observed that alcohol withdrawal ameliorated alcohol-induced hepatic steatosis by restoring lipid metabolic enzymes, and mitigated alcohol-induced liver injury and inflammation probably through TLR4/MAPKs involved signalling pathway. Our results enriched our understanding in the mechanisms of the beneficial roles of alcohol abstinence on ALD.

## Materials and Methods

### Ethics Statement

This study was carried out in strict accordance with recommendations from the Guide for the Care and Use of Laboratory Animals of the Chinese Association for Laboratory Animal Science. All animal care and protocols were approved by the Animal Care and Use Committee of Zhejiang Chinese Medical University (ZSLL-2017-150). All killings were performed under avertin anaesthesia, and efforts were made to minimise animal suffering.

### Animal Treatment

Male C57BL/6J mice (Beijing Vital River Laboratory Animal Technology Co., Ltd.) weighing 18.51 ± 1.21 g were group-housed in cages in a temperature-controlled vivarium (22 ± 2°C) and maintained on a 12-h light/dark cycle. After 2 weeks of adaption, the 8-weeks-old mice were randomly divided into four groups (*n* = 8 per group): pair-fed (PF) group, alcohol-fed (AF) group, abstinence control (PF-PF) group, and alcohol abstinence (AF-PF) group. Mice in the PF or AF group were fed with isocaloric control liquid diet or ethanol-containing Lieber-DeCarli diet for 4 weeks, respectively (Bioserv, Frenchtown, NJ, United States) (Dou et al., 2020). PF-PF and AF-PF mice were first fed with isocaloric control liquid diet or ethanol-containing Lieber-DeCarli diet for 4 weeks, respectively, and then fed with isocaloric control liquid diet for another 1 week. Food intake and body weight were recorded weekly. Plasma and liver tissues were harvested for further assays at the indicated time point.

### Plasma Analysis

Plasma alanine aminotransferase (ALT), triglyceride (TG), glycerol, and FFAs levels were determined by using commercial kits from Nanjing Jiancheng Bioengineering Institute (Nanjing, China) according to the manufacturer’s instructions.

### Oxidative Stress Markers

Liver malondialdehyde (MDA), superoxide dismutase (SOD), and glutathione peroxidase (GSH-Px) were measured with commercial kits based on enzymatic methods (Nanjing Jiancheng Bioengineering Institute, Nanjing, China).

### Histological Examination

Histological examination was performed as previously described (Li et al., 2020). In brief, small pieces of fresh liver were fixed immediately in 10% buffered formalin. After paraffin embedding, 5 μm sections were deparaffinised in xylene and rehydrated through a series of decreasing concentrations of ethanol. Sections were stained with hematoxylin and eosin (H & E) using a commercial kit (Nanjing Jiancheng Bioengineering Institute, Nanjing, China). Fat accumulation was examined by staining the liver sections with oil red O. Frozen liver sections were washed twice in phosphate buffered saline for 5 min. Oil redO and 85% propylene glycol were added with agitation for 15 min, and then the sections were washed with tap water. Stained sections were examined by light microscopy (Nikon, Ti-S, Japan).

### Western-Blot Analysis

Protein isolation from liver tissue and Western blot analysis were performed as previously described (Dou et al., 2018). The following antibodies were used: anti-phosphorylated-AMPKα (Thr172), anti-AMPKα, anti-PPARα, anti-CPT-1, anti-acetyl-CoA-carboxylase (ACC), anti-phosphorylated-ACC, anti-SREBP-1c, anti-fatty acid synthase (FAS), anti-DGAT-2, anti-P53, anti-Bcl2, anti-Bax, anti-TLR4, anti-JNK, anti-phosphorylated-JNK, anti-phosphorylated-P38, anti-P38, anti-phosphorylated-ERK, anti-ERK, anti-cleaved-caspase-3, and anti-GAPDH (Cell Signalling Technology, Danvers, MA, United States).

### Quantitative Real-Time Reverse Transcription Polymerase Chain Reaction

Total RNA extraction, reverse transcription, and real-time polymerase chain reaction were performed as previously described (Ma et al., 2019). Briefly, total RNA was extracted from liver samples using the TRIzol reagent. The primers are listed in Table 1 qRT-PCR was performed on an ABI 7300 PCR instrument using SYBR Green (Bimake, Houston, TX). Relative gene expression was calculated after normalisation by 18S.

**TABLE 1:** Primer sequence for quantitative real-time PCR.

Gene	Sequence
*18S*	F: 5′-AGG​TCT​GTG​ATG​CCC​TT-3′
	R: 5′-GAA​TGG​GGT​TCA​ACG​GGT​TA-3′
*TNF-α*	F: 5′-CCC​TCA​CAC​TCA​CAA​ACC​AC-3′
	R: 5′-ACA​AGG​TAC​AAC​CCA​TCG​GC-3′
*IL-6*	F: 5′-TGG​AAA​TGA​GAA​AAG​AGT​TGT​GC-3′
	R: 5′-CCA​GTT​TGG​TAG​CAT​CCA​TCA-3′
*IL-1β*	F: 5′-TTC​ATC​TTT​GAA​GAA​GAG​CCC​AT-3′
	R: 5′-TCG​GAG​CCT​GTA​GTG​CAG​TT-3′

### Statistical Analysis

Statistical analysis was performed using one-way analysis of variance (ANOVA) and followed by post-hoc test with Fisher’s least significant difference (LSD). Data were presented as means ± SD. Differences between each group were considered to be statistically significant at *p* < 0.05.

## Results

### Alcohol Withdrawal Ameliorates Alcohol-Induced Hepatic Steatosis and Liver Injury

**FIGURE 1:**
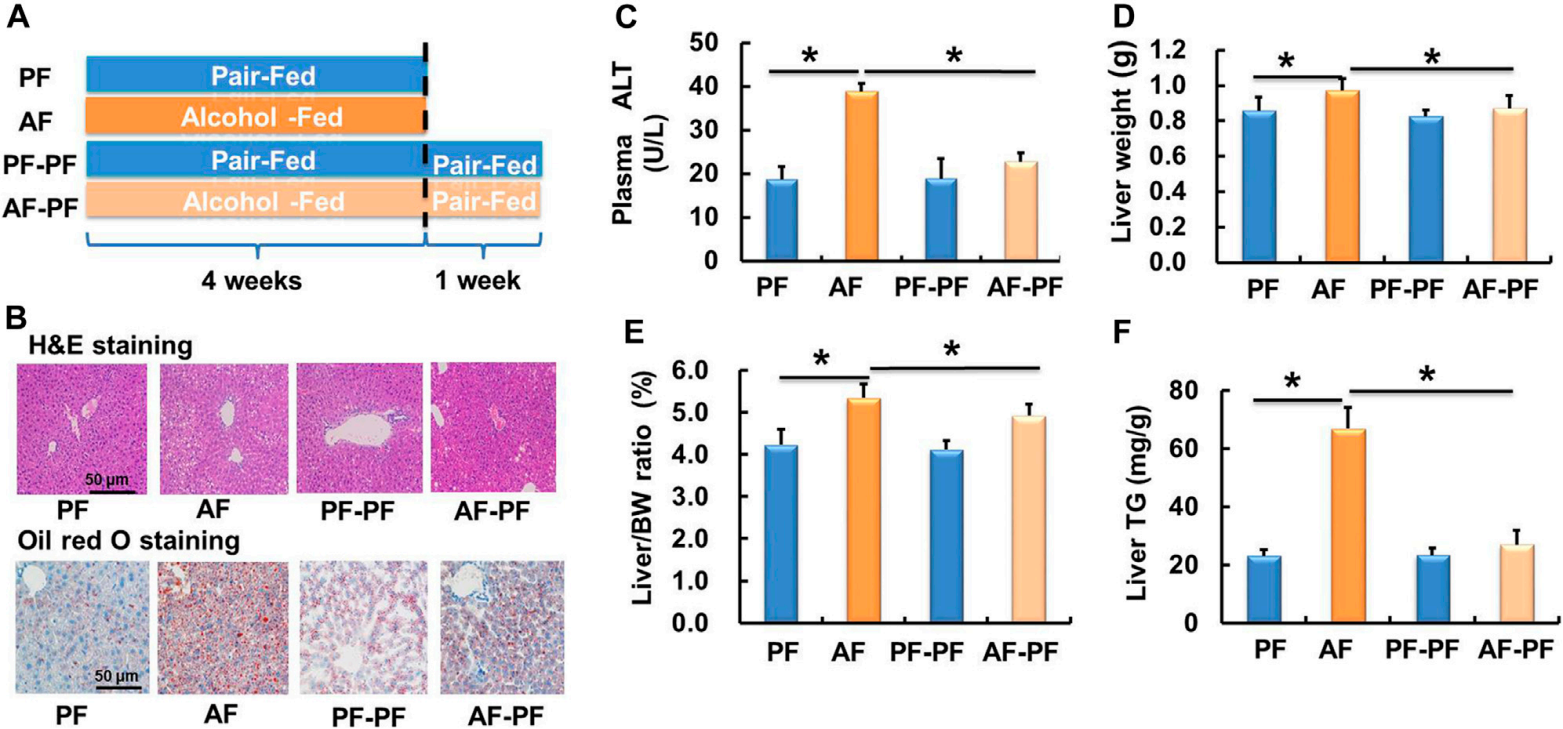
Alcohol withdrawal ameliorates alcohol-induced hepatic steatosis and liver injury. **(A)** Schematic diagram of the research design. **(B)** H & E staining and oil red O staining photomicrographs of the liver section (magnification, 100x). **(C)** Plasma alanine aminotransferase (ALT) level. **(D)** Live weight. **(E)** Liver weight/body weight ratio. **(F)** Triglyceride (TG) content in the liver. *p < 0.05 indicates statistically significant differences (*n* = 8).

A schematic diagram of the research design was shown in Figure 1A. The ALD mouse model was successfully established after 4 weeks of alcohol feeding, as evidenced by histological examinations (H & E and Oil Red O staining), plasma ALT, liver weight, liver weight/body weight ratio, and hepatic TG content (Figure 1). Strikingly, the alcohol-induced detrimental alterations mentioned above were strongly rescued after 1-week alcohol abstinence treatment (Figures 1B–E). Moreover, alcohol withdrawal also markedly reversed the hepatic TG accumulation-induced by alcohol-fed (Figure 1F). The body weight and food intake showed no significant difference among four groups (Supplementary Figures S1A,B).

### Alcohol Withdrawal Alleviates Alcohol-Induced Dyslipidaemia

**FIGURE 2:**
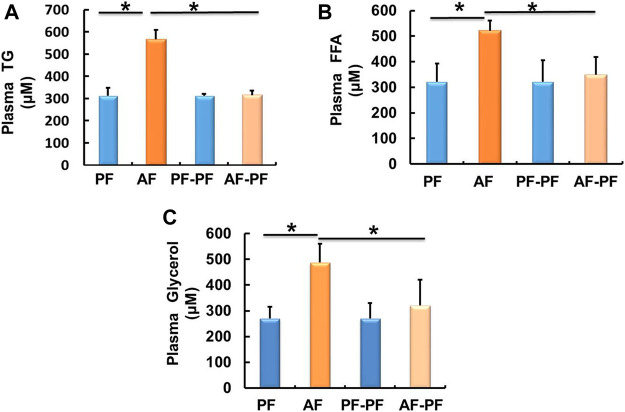
Alcohol withdrawal alleviates alcohol-induced dyslipidaemia. **(A)** Plasma triglyceride (TG) level. **(B)** Plasma FFA level. **(C)** Plasma glycerol level. Data are described as means ± SD from 8 mice in each group. *p < 0.05 indicates statistically significant differences (*n* = 8).

We subsequently detected lipids levels in animal blood. Our data showed that chronic alcohol exposure significantly elevated plasma TG and FFAs levels (Figures 2A,B). The glycerol content was also increased in the plasma of AF mice (Figure 2C). Alcohol withdrawal apparently reversed alcohol-induced hyperlipidaemia, as evidenced by the recovery of plasma TG, FFAs, and glycerol levels (Figure 2).

### Alcohol Withdrawal Improves the Activity of Antioxidant Enzymes in the Liver

Oxidative stress caused by alcohol metabolism is an important pathological mechanism of ALD. Therefore, we measured the levels of oxidative stress products and the activities of antioxidant enzymes in the liver. The results showed that 1 week alcohol withdrawal was not sufficient to reverse alcohol-induced excessive formation of MDA in the liver (Table 2). However, the activities of antioxidant enzymes, including SOD and GSH-px was rescued by alcohol withdrawal (Table 2).

**TABLE 2:** Oxidative stress markers level in liver tissue sample after alcohol withdrawal (*n* = 8).

	PF	AF	PF-PF	AF-PF
MDA (nmol/mg protein)	8.265 ± 1.16^a^	10.295 ± 2.03^b^	7.25 ± 1.59^a^	11.31 ± 2.46^b^
SOD (U/mg protein)	276.27 ± 31.65^a^	202.52 ± 24.19^b^	279.48 ± 42.64^a^	275.78 ± 45.09^a^
GSH-PX (U/mg protein)	1107.89 ± 122.63^a^	785.16 ± 63.85^b^	1109.30 ± 109.53^a^	1359.38 ± 185.30^c^

PF. pair-fed group; AF, alcohol-fed group; PF-PF, abstinence control group, and AF-PF, alcohol abstinence group. Different letters represent statistical differences (p < 0.05).

### Abstinence Improves the Expressions of Lipid Metabolism Related Enzymes in the Liver

**FIGURE 3:**
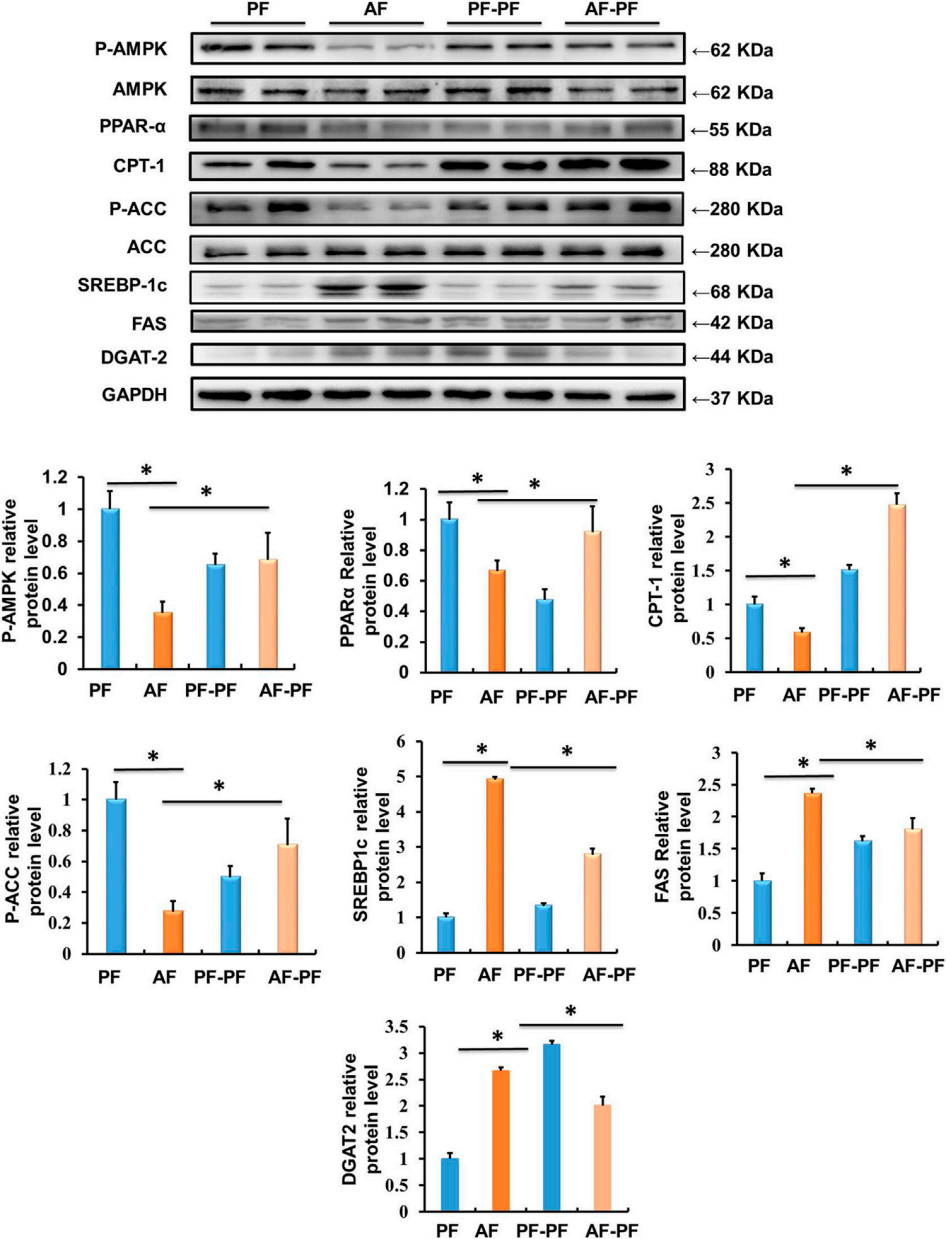
Alcohol withdrawal improves proteins related to liver lipid metabolism. Total cellular lysates were extracted from the liver samples. Western blot was performed to detect the expressions of p-AMPK, PPAR-α, CPT-1, p-ACC, mature-SREBP-1c, FAS, and DGAT-2. *p < 0.05 indicates statistically significant differences (*n* = 8).

Long-term alcohol intake leads to hepatic lipid accumulation by regulating the metabolic reprogramming of lipid metabolism related genes and regulatory factors in the liver. Correcting the abnormal expression of these genes is helpful to improve hepatic steatosis. Accordingly, we assessed the effects of alcohol withdrawal on the protein expressions of various signalling molecules involved in lipid metabolism in liver tissues. As shown in Figure 3, key proteins in the regulating of lipid catabolism, including phosphorylated-AMPKα, PPARα, and CPT-1 were significantly down-regulated by alcohol-fed. Meanwhile, the proteins, which control the *de novo* synthesis of fatty acids, such as mature-SREBP-1c and phosphorylated-ACC were activated by chronic alcohol intake (Figure 3). Alcohol feeding also promoted the expression of DGAT2, which is a rate-limiting enzyme in the regulation of triglycerides synthesis (Figure 3). Notably, alcohol abstinence markedly reversed the expression of above proteins induced by alcohol (Figure 3), implying that alcohol withdraw is an effective way to recover the lipid metabolism reprogramming in the liver.

### Alcohol Withdrawal Rescues Alcohol-Stimulated TLR4/MAPKs and Mitochondrial Apoptotic Pathways

**FIGURE 4:**
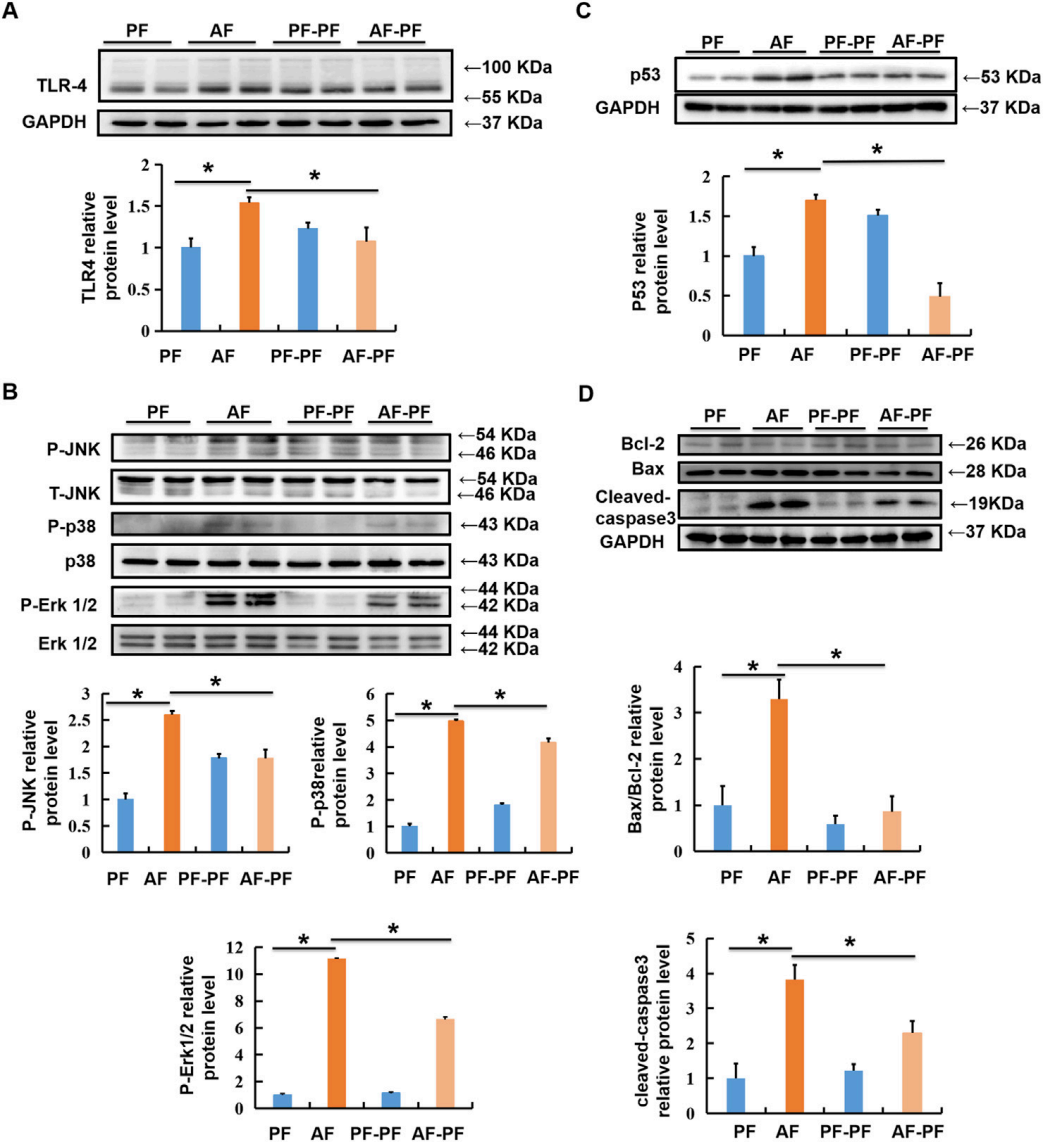
Alcohol withdrawal rescues alcohol-stimulated TLR4/MAPKs and mitochondrial apoptotic pathways. Total cellular lysates were extracted from mice liver tissues. **(A, B)** Immunoblotting assay was performed for TLR-4, p-JNK, p-P38, and p-ERK1/2. **(C,D)** Immunoblotting assay was performed for P53 and Bax/Bcl-2. *p < 0.05 indicates statistically significant differences (*n* = 8).

Hepatic lipid accumulation induced by chronic alcohol intake, which in turn leads to lipotoxicity and further hepatocyte death, is a critical pathological mechanism in ALD. Previous studies including ours have indicated that TLR4/MAPKs pathway was mechanistically involved in alcohol- and lipotoxicity-induced hepatotoxicity [(Shen et al., 2018; Li et al., 2020; Yang et al., 2021)]. Here, we evaluated the beneficial effect of abstinence on TLR4/MAPKs pathway. Our data clearly revealed that alcohol exposure significantly stimulated the expression of TLR4 in the liver, accompanied with MAPKs activation, evidenced by the increases in phosphorylation of JNK, p38, and ERK1/2 (Figures 4A,B). Alcohol withdrawal robustly rescued alcohol-induced activation of TLR4/MAPKs pathway (Figures 4A,B). Moreover, alcohol-increased expression of pro-apoptotic protein p53, which is a well-known target of MAPKs, was also reduced by abstinence (Figure 4C). We subsequently detected the expressions of marker proteins for mitochondrial apoptotic pathway, and observed that alcohol-elevated Bax/Bcl-2 ratio and cleaved-caspase-3 were reversed by alcohol withdrawal (Figure 4D).

### Alcohol Withdrawal Mitigates Alcohol-Induced Hepatic Inflammation

**FIGURE 5:**
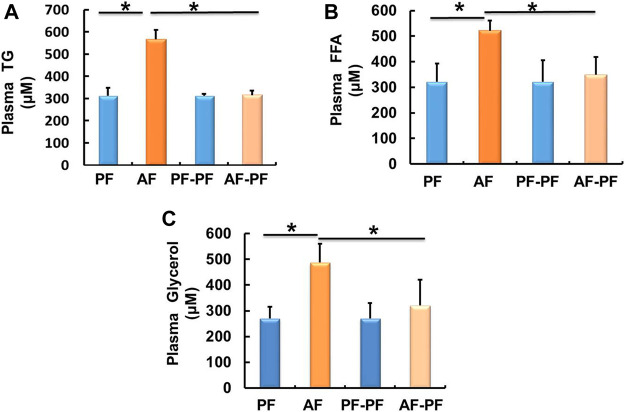
Alcohol withdrawal mitigates alcohol-induced hepatic inflammation. **(A)** The mRNA levels of *TNF-α* **(A)**, *IL-6* **(B)**, and *IL-1β* **(C)** in the liver. ^*^ *p* < 0.05 indicates statistically significant differences (n = 6−8).

Previous studies have identified several proinflammatory factorsplayed important roles in the pathogenesis of ALD (Gao and Tsukamoto, 2016; Xu et al., 2017). In the present study, we observed that chronic alcohol feeding markedly stimulated the expressions of *TNF-α* and *IL-6 a*t the transcriptional level, whereas alcohol withdrawal effectively reversed such detrimental alteration (Figure 5). We did not observe any statistically significant differences in the mRNA expression of IL-1β among the four groups (Figure 5C), probably due to the large variations and small changes.

## Discussion

Alcohol-induced liver metabolic reprogramming is an important pathological basis of ALD. Cessation of alcohol consumption is the most crucial prerequisite of therapy for ALD (Leggio and Lee, 2017). Alcohol abstinence reduces the risk of ALD progression and even has a statistically significant impact on survival (Addolorato et al., 2016). However, the potential mechanisms through which alcohol withdrawal improves reprogramming and further hepatic steatosis, and even liver injury are not fully understood. In this study, we provided strong evidence that 1 week of alcohol withdrawal reversed alcohol-induced alterations in lipid metabolism, oxidative stress, inflammation, and liver injury in mice. Further mechanistic study revealed that liver lipid metabolism reprogramming was corrected by alcohol abstinence. Alcohol withdrawal restored the enzymes and regulatory factors in promoting FFAs oxidation and inhibiting FFAs and TG synthesis. Furthermore, the inhibition of TLR4/MAPKs pathway might also contribute to the beneficial role of alcohol withdrawal. Our study demonstrated that alcohol abstinence improved alcohol-mediated metabolic reprogramming by regulating the expression of multiple metabolic related proteins, however, the detection of the activities of these key proteins will be more helpful to reveal the biological mechanism in this process, which should be investigated in further studies.

Feeding rodents with the Lieber-DeCarli ethanol liquid diet caused fat accumulation and mild liver injury (Arteel, 2010). Consistent with previous reports, an AFL mouse model was successfully established by feeding mice with a Lieber-DeCarli ethanol liquid diet for 4 weeks. Previous studies have reported that 1 week of abstinence was adequate and appropriate for mice to recover from AFL after a 4-weeks drinking history (Casey et al., 1989; Thomes et al., 2019). Therefore, 1 week abstinence was selected in the design of this study. Consistent with previous studies, we observed that 1 week of alcohol withdrawal significantly reversed hepatic steatosis and liver injury caused by alcohol feeding.

Malnutrition caused by chronic alcohol consumption was initially believed to contribute to the development of ALD. However, the discovery of ROS generation with ethanol catabolism changes this dogma (Lu and Cederbaum, 2018). Excessive ROS-stimulated oxidative stress has been considered playing a vital role in the pathological process of ALD. In the state of oxidative stress, MDA is generated from lipid peroxidation, and binds to DNA bases and causes liver injury (Linhart et al., 2014; Mueller et al., 2018). Moreover, excessive ethanol intake exhausts hepatic capacity of endogenous antioxidants including GSH and SOD (Leung and Nieto, 2013). In this study, we detected the protective role of alcohol withdrawal on oxidative stress in the liver. Unexpectedly, our data indicated that only 1 week abstinence was not enough to completely reverse alcohol-increased MDA content in liver. However, alcohol withdrawal rescued the activity of hepatic antioxidant enzymes, including SOD and GSH-PX. Further study should be focused on whether extending abstinence and how long the extension will be required to help eliminating the increased hepatic MDA.

Oxidative stress promotes reprogramming of liver genes, which are in charge of lipid metabolism, programmed apoptosis, and inflammation, and further induces hepatic steatosis, liver injury, and hepatitis (Ceni et al., 2014). Recently, Paul et al. found that alcohol withdrawal attenuated alcohol-induced hepatic steatosis and injury by normalizing aberrant fatty acid transport and restoring lysosomal function in rats (Thomes et al., 2019). In the present study, we evaluated the beneficial effect of alcohol abstinence on the key enzymes or regulators of lipid catabolism and synthesis. AMPK acts as a central signal switch that controls lipid metabolism pathways (Winder and Hardie, 1999; You et al., 2004). Chronic alcohol exposure inhibited hepatic phosphorylated-AMPK expression. However, drug induced activation of AMPK or AMPK overexpression significantly improved alcohol-induced hepatic steatosis and liver injury (Nagappan et al., 2019; Silva et al., 2021). In this study, our data indicated that the phosphorylation of AMPK was inhibited by 4-weeks alcohol exposure, which was restored by alcohol withdrawal. CPT-1, a downstream target of AMPK, is a rate-limiting enzyme in FFA β-oxidation (Ronnett et al., 2006). Previous report showed that ethanol decreased the activity of CPT-1 (You et al., 2004). In the present study, we observed that alcohol withdrawal rescued the reduction of CPT-1. The role of PPARα in fatty liver disease has been investigated in the past decade. PPARα is a nuclear hormone receptor that controls the transcription of a number of genes involved in FFA transport and oxidation (Galli et al., 2001). Excessive alcohol consumption leads to the downregulation of PPARα and its target genes, which inhibits the β-oxidation of fatty acids (Nakajima et al., 2004). In mouse models of ALD, PPARα ligands treatment restored receptor activity and significantly ameliorated fat accumulation (Fischer et al., 2003; Nanji et al., 2004). Consistent with previous reports, the expression of PPARα was inhibited by alcohol but was upregulated with alcohol withdrawal. These findings suggested that alcohol abstinence improves lipid accumulation in the liver by reprogramming key enzymes and regulatory factors of fatty acid catabolism.

In the initial stage of ALD, the imbalance between FA synthesis and metabolism contributed to hepatic steatosis (Purohit et al., 2009). SREBP-1c is a critical factor regulating the *de novo* lipogenesis. Mature SREBP-1c (the active form) promotes fatty acid biosynthesis by upregulating the expression of lipogenesis-related genes, including ACC, FAS and DGAT-2 (You et al., 2002). The expression of hepatic SREBP-1c was increased by alcohol exposure (Ji and Kaplowitz, 2003; Hu et al., 2012). Consistent with previous studies, chronic alcohol exposure significantly increased the expression of hepatic mature-SREBP-1c, FAS, and DGAT-2, and inhibited the phosphorylation of ACC. Importantly, those alterations were reversed by alcohol withdrawal. These results suggested that alcohol abstinence improved chronic alcohol exposure-induced hepatic steatosis *via* decreasing the *de novo* lipogenesis.

Lipotoxicity is a vital pathological factor in ALD. Previous studies showed that TLR4/MAPKs pathway was mechanistically involved in lipotoxicity-induced hepatotoxicity (Shen et al., 2018). TLR4 activation-stimulated MAPKs pathway was mechanistically implicated in ALD (Fitzgerald and Kagan, 2020). While, TLR4 deficiency protected against alcohol-induced steatosis by protecting against inflammatory cytokines production (Uesugi et al., 2001; Hritz et al., 2008). In our study, alcohol withdrawal effectively reversed alcohol-induced phosphorylated-p38, -ERK1/2 and -JNK. P53, a downstream target of MAPKs, was also reversed by alcohol withdrawal. We noticed that the expressions of p-JNK and Bax/Bcl-2 were returned to the same levels of the control group after alcohol withdrawal. However, some proteins, such as p38, pERK1/2, and cleaved-caspase -3 were only partially reversed. we speculated that this was due to the short duration of abstinence. After alcohol treatment, TLR4 recruits complicated adaptor proteins to initiate MyD88 pathways, which transactivate the transcription and secretion of the gene for TNF-α, an inflammatory cytokine (Gustot et al., 2006). In the present study, decreased level of inflammatory cytokine, TNF-α and IL-6, were observed after alcohol withdrawal. The existing evidence indicates that alcohol withdrawal rescues alcohol-stimulated TLR4/MAPKs and mitigates alcohol-induced hepatic inflammation.

Mitochondrial dysfunction has been detected in both experimental animal and patients with ALD (Yacoub et al., 1995; Ziol et al., 2001). Chronic alcohol consumption promoted programmed apoptosis mediated by mitochondria *via* upregulating Bax and down-regulating Bcl-2, leading to mitochondrial permeabilization increase, release of cytochrome c and activation of caspases 3 (Guo et al., 2009). In this study, we confirmed that 1-week alcohol abstinence strongly reversed alcohol-fed induced mitochondrial dysfunction, evidenced by the observation of decreased cleaved-caspase3 expression and increased Bax/Bcl-2 ratio.

Taken together, alcohol withdrawal effectively reversed chronic alcohol intake-caused liver metabolic reprogramming, and in hence improved hepatic steatosis, liver injury, and inflammation. Our findings provide a theoretical basis for the understanding of alcohol withdrawal on ALD recovery.

## Data Availability

The original contributions presented in the study are included in the article/Supplementary Material, further inquiries can be directed to the corresponding authors.
