# Corrigendum: Alcohol abstinence rescues hepatic steatosis and liver injury *via* improving metabolic reprogramming in chronic alcohol-fed mice

**DOI:** 10.3389/fphar.2025.1554390

**Published:** 2025-02-19

**Authors:** Aiwen Pi, Kai Jiang, Qinchao Ding, Shanglei Lai, Wenwen Yang, Jinyan Zhu, Rui Guo, Yibin Fan, Linfeng Chi, Songtao Li

**Affiliations:** ^1^ School of Public Health, Zhejiang Chinese Medical University, Hangzhou, China; ^2^ School of Life Science, Zhejiang Chinese Medical University, Hangzhou, China; ^3^ Academy of Chinese Medical Science, Zhejiang Chinese Medical University, Hangzhou, China; ^4^ Molecular Medicine Institute, Zhejiang Chinese Medical University, Hangzhou, China; ^5^ Department of Dermatology, People’s Hospital of Hangzhou Medical College, Zhejiang Provincial People’s Hospital, Hangzhou, China; ^6^ School of Basic Medicine, Zhejiang Chinese Medical University, Hangzhou, China

**Keywords:** alcoholic liver disease, alcohol abstinence, hepatic steatosis, liver injury, hepatic inflammation

In the published article, there was an error in Figure 4 as published. The statistical analysis column chart in Figure 4B was based on the ratio of phosphorylation target proteins to non-phosphorylation of target proteins, but not to internal reference protein GAPDH. To avoid any potential misunderstanding, the band of GAPDH was removed from Figure 4B, as it is unrelated to the quantification of detected proteins.

**FIGURE 4:**
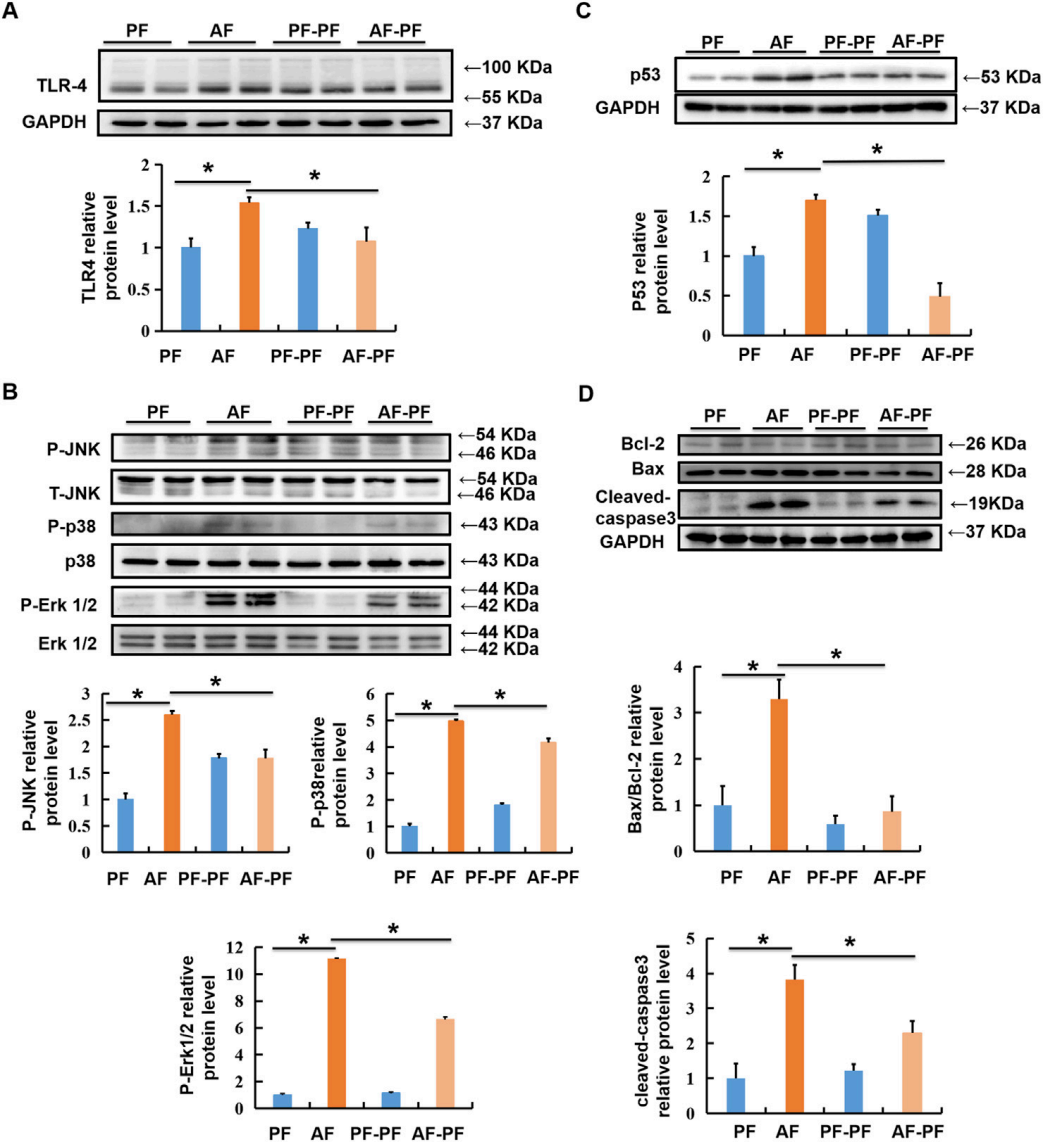
Alcohol withdrawal rescues alcohol-stimulated TLR4/MAPKs and mitochondrial apoptotic pathways. Total cellular lysates were extracted from mice liver tissues. **(A, B)** Immunoblotting assay was performed for TLR-4, p-JNK, p-P38, and p-ERK1/2. **(C, D)** Immunoblotting assay was performed for P53 and Bax/Bcl-2. *p < 0.05 indicates statistically significant differences (n = 8).

Additionally, the same band of GAPDH was used both in Figures 4C, D, since the bands and data in those panels were based on the same samples with the same loading amount. In order to avoid confusion, we have replaced the band in Figure 4C with other representative images selected from our research records. In the original version, the statistical analysis of data was performed based on all the bands, including previously and currently displayed, therefore, this replacement does not affect the results and conclusions. The corrected Figure 4 and its caption appear below.

‘Alcohol withdrawal rescues alcohol-stimulated TLR4/MAPKs and mitochondrial apoptotic pathways. Total cellular lysates were extracted from mice liver tissues. (A, B) Immunoblotting assay was performed for TLR-4, p-JNK, p-P38, and p-ERK1/2. (C, D) Immunoblotting assay was performed for P53 and Bax/Bcl-2. *p < 0.05 indicates statistically significant differences (n = 8).’

The authors apologize for this error and state that this does not change the scientific conclusions of the article in any way. The original article has been updated.

