# Supplementary figures and images for: Alcohol Abstinence Rescues Hepatic Steatosis and Liver Injury via Improving Metabolic Reprogramming in Chronic Alcohol-Fed Mice
Source: Front Pharmacol. 2021 Sep 16;12:752148. doi: 10.3389/fphar.2021.752148 (PMC8481816; doi:10.3389/fphar.2021.752148)

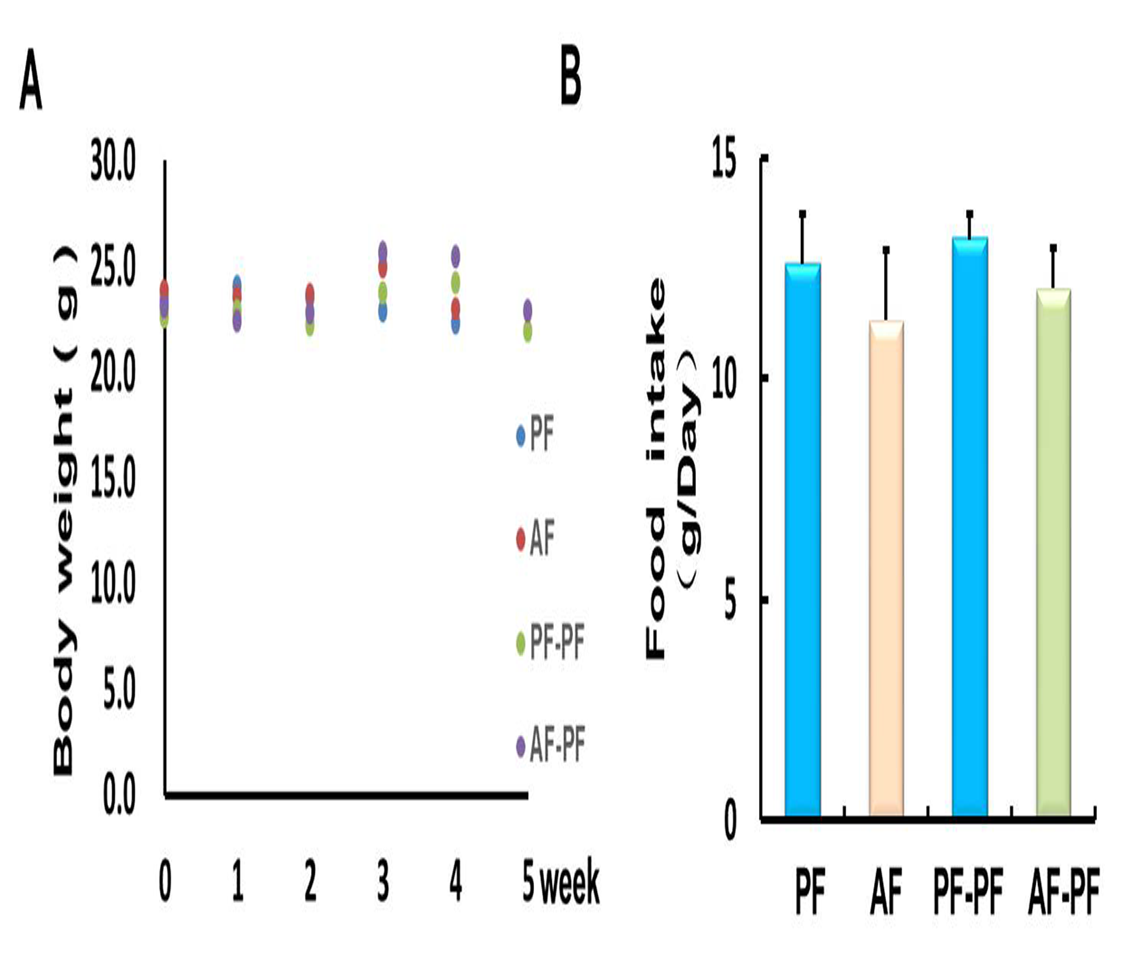

Supplement: Supplementary file 1 [file Image1.TIF]

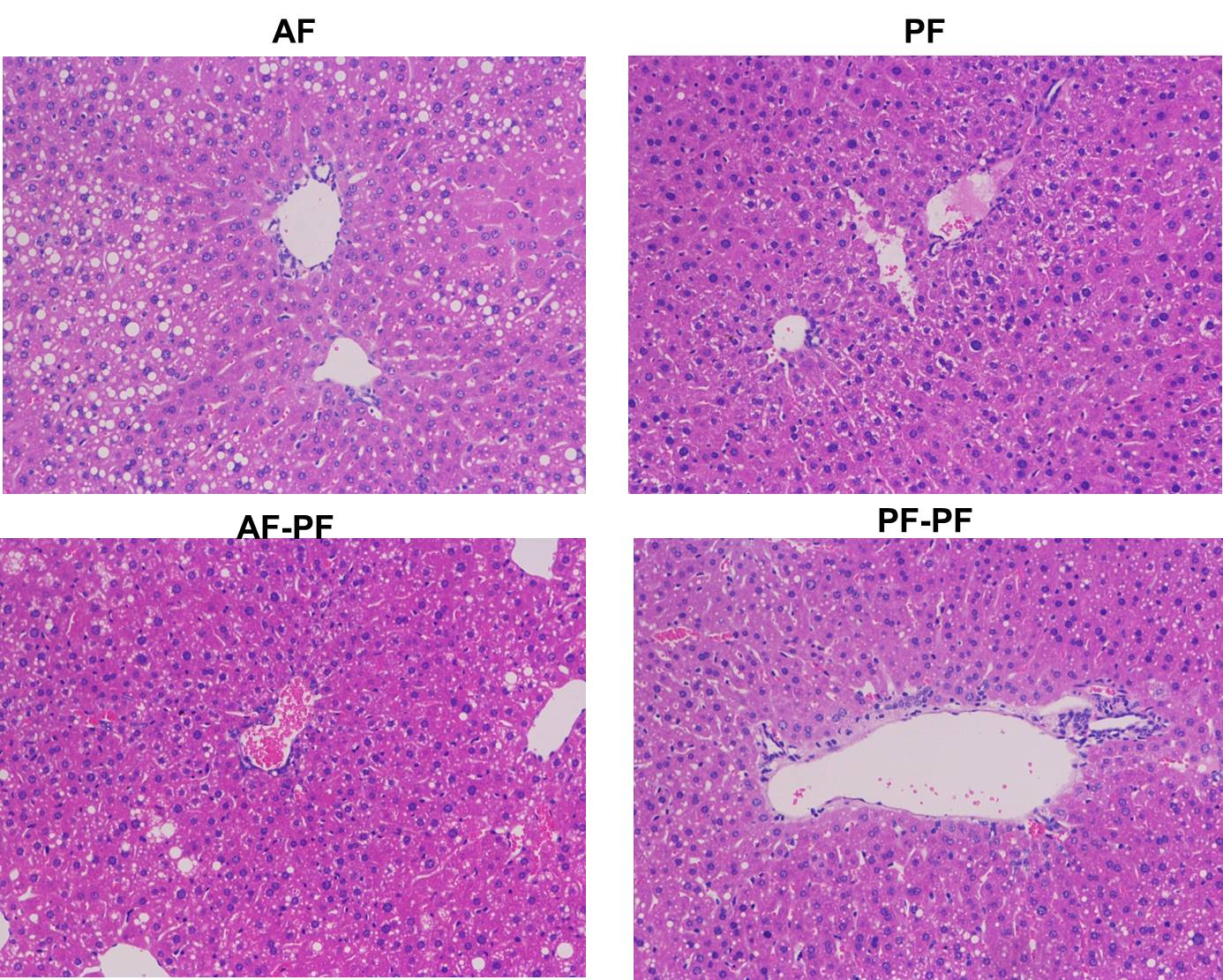


Figure 1B


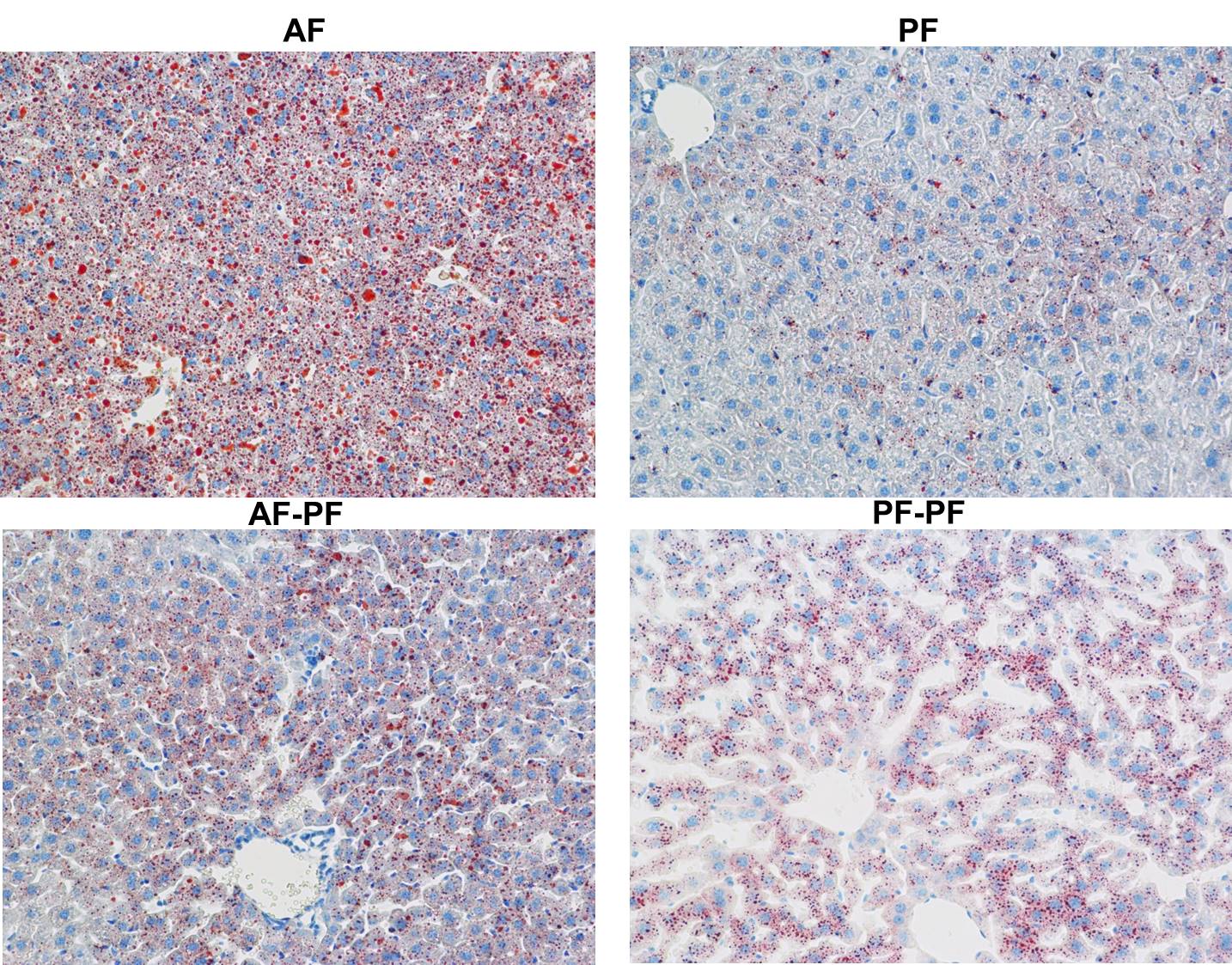


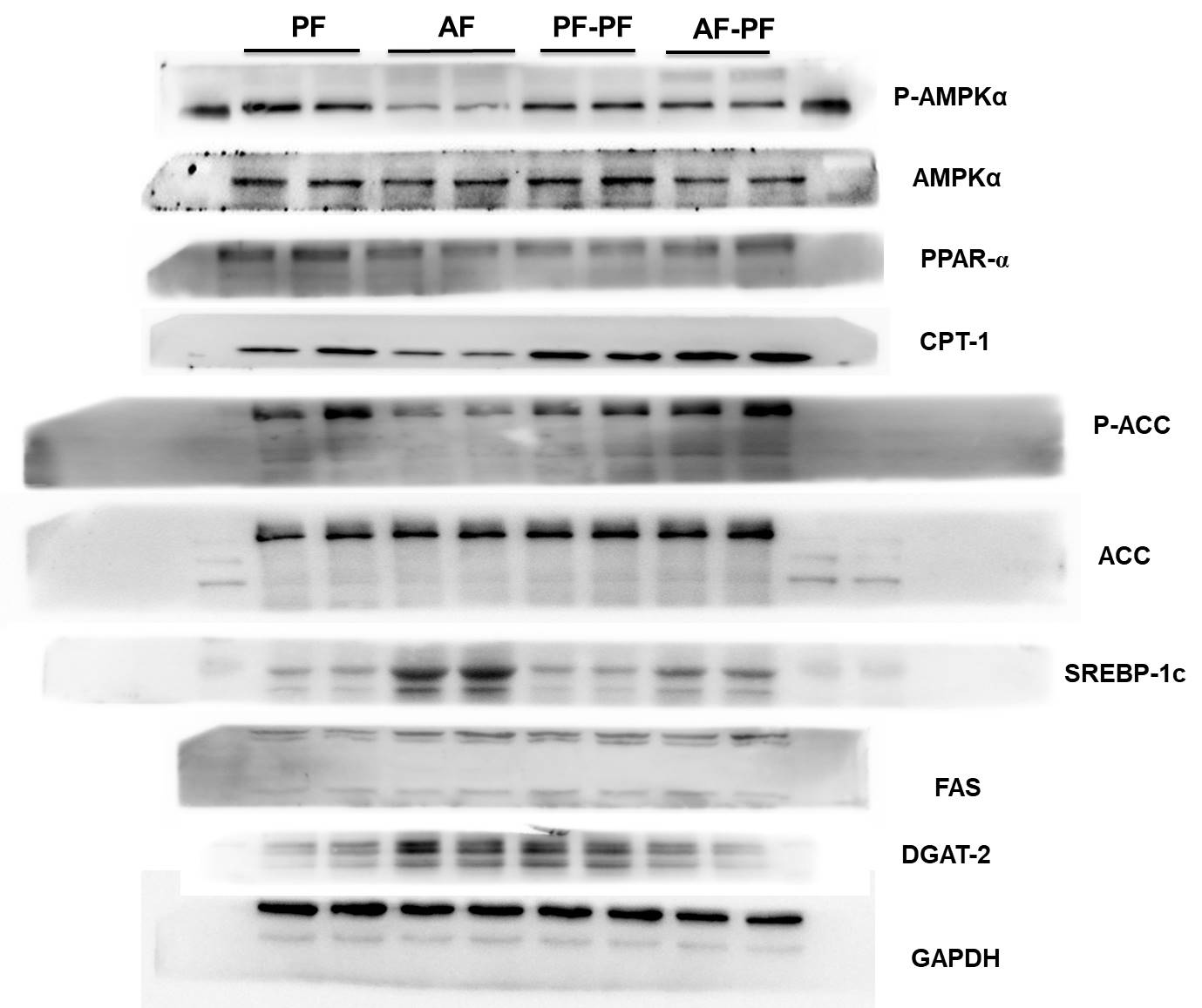


Figure 3


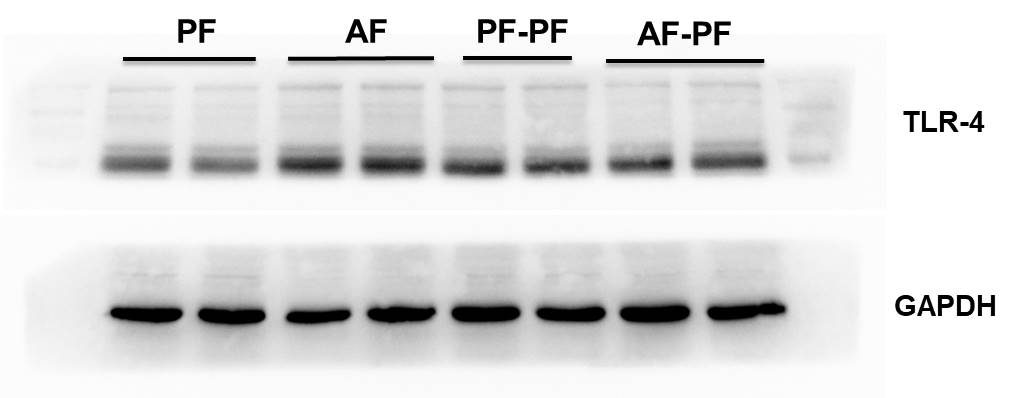


Figure 4A

Figure 4B


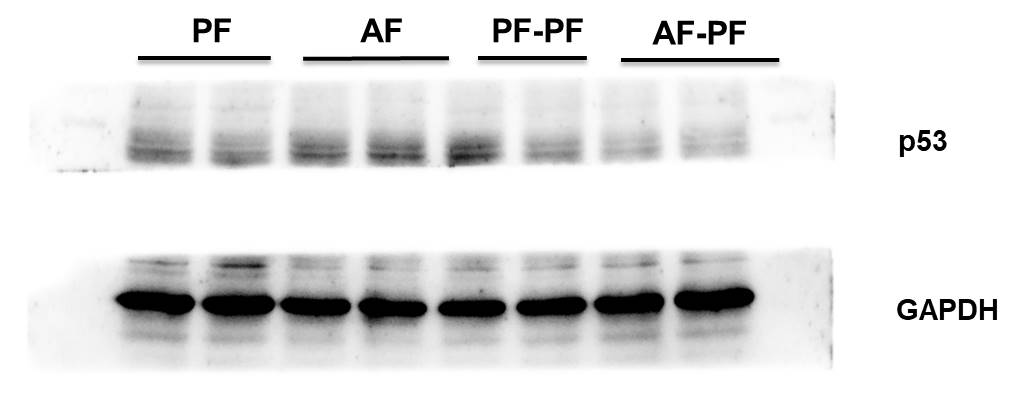


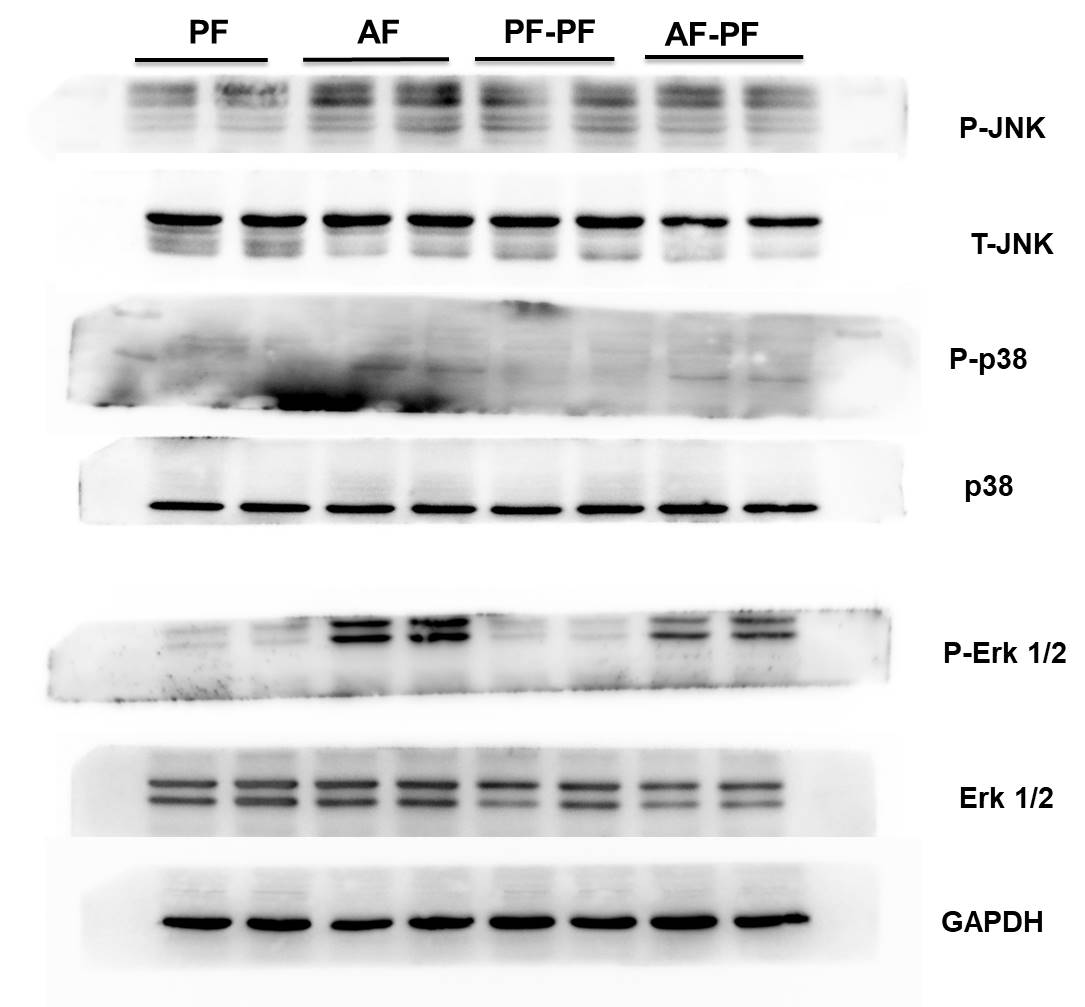


Figure 4D

Figure 4C


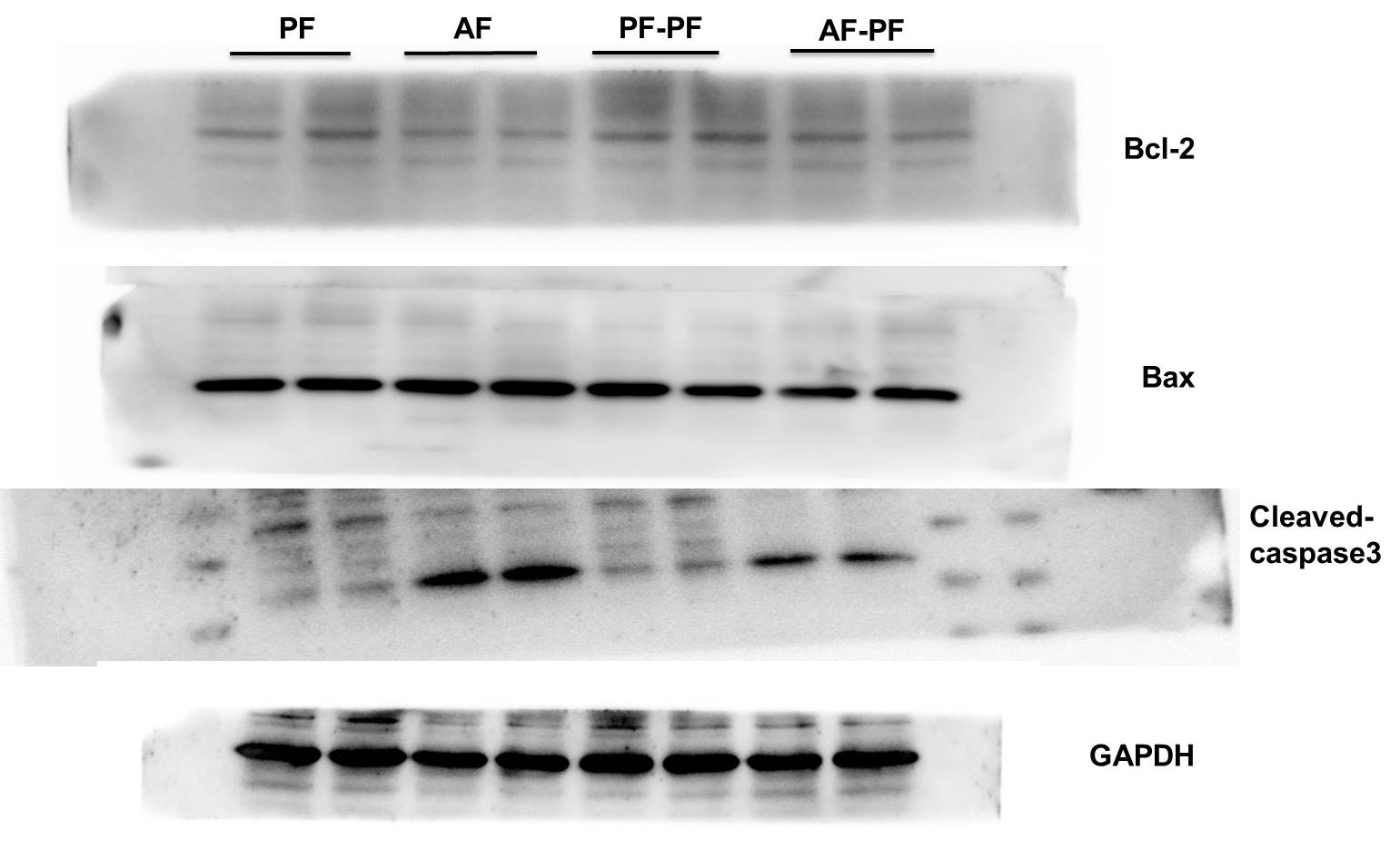

Supplement: Supplementary file 2 [file DataSheet1.docx]
